# Multifunctionality and Possible Medical Application of the BPC 157 Peptide—Literature and Patent Review

**DOI:** 10.3390/ph18020185

**Published:** 2025-01-30

**Authors:** Michalina Józwiak, Marta Bauer, Wojciech Kamysz, Patrycja Kleczkowska

**Affiliations:** 1 Maria Sklodowska-Curie Medical Academy in Warsaw, 03-411 Warsaw, Poland; michalina1395@interia.pl; 2 Department of Analytical Chemistry, Faculty of Pharmacy, Medical University of Gdansk, 80-416 Gdansk, Poland; 3 Department of Inorganic Chemistry, Faculty of Pharmacy, Medical University of Gdansk, 80-416 Gdansk, Poland; wojciech.kamysz@gumed.edu.pl

**Keywords:** BPC 157, molecular pathways, efficacy and application, safety, BCP 157-based patents

## Abstract

BPC 157, known as the “Body Protection Compound”, is a pentadecapeptide isolated from human gastric juice that demonstrated its pleiotropic beneficial effects in various preclinical models mimicking medical conditions, such as tissue injury, inflammatory bowel disease, or even CNS disorders. Unlike many other drugs, BPC 157 has a desirable safety profile, since only a few side effects have been reported following its administration. Nevertheless, this compound was temporarily banned by the World Anti-Doping Agency (WADA) in 2022 (it is not currently listed as banned by the WADA). However, it has not been approved for use in standard medicine by the FDA and other global regulatory authorities due to the absence of sufficient and comprehensive clinical studies confirming its health benefits in humans. In this review, we summarize information on the biological activities of BPC 157, with particular reference to its mechanism of action and probable toxicity. This generated the attention of experts, as BPC 157 has been offered for sale on many websites. We also present recent interest in BPC 157 as reflected in a number of patent applications and granted patents.

## 1. Introduction

Despite considerable technological developments and continuous research to find and implement an effective and safe drug, there are still none that have positive therapeutic effects, target multiple disease entities, and, at the same time, have no side effects.

Currently, much effort has been expended on naturally occurring compounds, especially peptides, as they can serve as a powerful model on the basis of which researchers develop new effective tools similar to natural ones to control various physiological mechanisms [1]. Indeed, many important functions in the body are based on biological properties of peptide molecules. These include hormonal, neurotransmitting, and immunomodulatory functions [2]. Normal protein degradation and metabolic processes also depend on them. In addition, they have satisfactory curative effects and no obvious side effects. The advantage of this type of compounds, apart from the reduction in side effects, is their efficient metabolism to non-toxic products and a high affinity for specific molecular targets, which can potentially be associated with enhanced treatment efficacy, enabling the use of a relatively low dose to produce identical biological effects [3]. Another undisputed advantage of peptide drugs is also the absence of accumulation in the body, as well as the fact that peptides show enormous chemical and biological diversity. Therefore, their use together with a slight modification of the parent compound’s structure can lead to the desired therapeutic profile, including a low risk of serious clinically important side effects. However, compounds with an amino acid-based structure (i.e., peptides, proteins, peptidomimetics), including those used in therapeutic applications, have a number of serious limitations, such as conformational instability, short duration of action (short half-life), and insufficient penetration through the blood–brain barrier (BBB) [3,4,5]. In addition, most of these molecules are characterized by rapid hepatic and renal clearance and inadequate passive transport across cell membranes. All of the above characteristics contribute to the reduced absorption and distribution of the drug in the body [6,7]. In addition, the absence of resistance of peptides to breakdown by gastrointestinal proteolytic enzymes (e.g., carboxypeptidases or aminopeptidases) should be mentioned [8], which in practice precludes administrating such a drug to the patient orally and calls for intravenous administration.

BPC 157, also known as PLD-116, PL-10, PL14736 [9], or Bepectin [10,11,12,13,14,15,16,17], has recently attracted considerable attention as a potential drug candidate that is stable in acidic environments and has no serious adverse effects [18], although it has been known to cause pain and/or necrosis when injected in an aqueous solution or in physiological saline [19]. Furthermore, no toxic dose has been determined to date. In fact, in a toxicity study in Sprague-Dawley rats conducted with a single dose of 20 mg/kg (intramuscular, i.m.) of pentadecapeptide, no deaths or obvious abnormalities in body weight, food intake, or behavior were reported. Studies with beagle dogs at a dose of 10 mg/kg i.m. [18] also showed no distinct adverse effects of the substance. A 28-day administration of BPC 157 (4, 1, or 0.2 mg/kg/day, i.m.) in rats and (2, 0.5, or 0.1 mg/kg/day) in beagle dogs also resulted in no apparent changes as compared to saline-treated animals. Interestingly, it was found that this agent has a highly protective effect against adverse effects mediated by various medical agents, such as non-steroidal anti-inflammatory drugs (NSAIDs) and alcohol [20,21,22,23]. Also, its healing actions on wounds and injuries, both traumatic and systemic, were evident [24,25]. Apart from the above-mentioned activities, BPC 157 has shown further beneficial effects in neuropsychiatric conditions, such as depression [26].

This paper aims at presenting the BPC 157-induced therapeutic effects, which have been confirmed in several reports, but also to address probable side effects that may, however, result from the mechanism of action and the activation of signaling pathways by the peptide.

## 2. BPC 157 Characteristics—Structural Analysis, Targeted Receptors/Molecular Pathways, and ADME Profile

BPC 157 is a pentadecapeptide with the amino acid sequence of Gly-Glu-Pro-Pro-Pro-Gly-Lys-Pro-Ala-Asp-Asp-Ala-Gly-Leu-Val and a molecular weight of 1419.55 daltons, which was first introduced and described by Sikiric and colleagues in 1993 [27]. Although it was isolated as part of the peptide from human gastric juice [28], it shows no sequence homology with known intestinal peptides [29]. Remarkably, this specific sequence is considered to be essential and fully responsible for biological activities of the compound [30]. So, there are only a few studies aimed at determining the beneficial properties of the modified analogues of BPC 157 [31]. Interestingly, such a structural composition renders this peptide stable in water and gastric juice [32]. This property is important because many peptide-based molecules have been known for their low stability [33], hence frequent administrations are usually required to ensure the efficacy of the drug, especially when delivered orally. Nevertheless, the specific amino acid composition and sequence of the peptide determine its physicochemical characteristics as well as its pharmacokinetics and pharmacodynamics. In this context, factors such as charge state, hydrophobicity, and other features are important.

In view of this, various strategies to improve peptide stability have been presented, including modifications of the C/N-terminus [34] and *D*-amino acid substitution [35]. The latter can occur naturally, such as the opioid peptide dermorphin isolated from frog skin, peptidoglycans of some microorganisms, or venom peptides—conotoxins containing *D*-tryptophan (*D*-Trp), *D*-leucine (*D*-Leu), etc. [36]. However, in the case of the BPC 157 peptide, the above-mentioned structural features have not been reported. Therefore, a detailed analysis of the sequence is recommended.

In this context, Xu et al. [37] have recently demonstrated that the N-terminally located glycine (Gly), present in the BPC 157 pentedecapeptide, can serve as a stabilizer that can regulate the compound’s protease degradation [37]. Similarly, proline (Pro), known to exist either in *cis* or *trans* isomers, has been reported to affect protein folding [38]. This, in turn, affects compound stability [39]. In addition, it has been shown that repeating proline motifs within peptide chains (with BPC 157, triple proline motifs occur) play a crucial role in the protection. In fact, it was found that proline residues located one after another in a row strongly prevent the so-called non-specific proteolysis [40]. In contrast, peptides containing asparagine (Asp) are more susceptible to dehydration.

As BPC 157 offers a number of beneficial effects, several studies were carried out to gain information on the probable mechanism by which the effects are mediated. Most studies identify the pentadecapeptide as a compound that interacts with the nitric oxide (NO) system [23,41]. In this context, it has been found to counteract the deleterious effects of NG-nitro-L-arginine methyl ester (L-NAME), such as an ulcerogenic effect, and some of L-arginine [42,43,44]. In addition, BPC 157 plays a role in oxidative stress and exhibits a strong antioxidant activity through its ability to stabilize free radical scavengers or counteract free radical formation and lesions [45,46,47]. This, in turn, could testify in favor of the peptide’s suspected positive effect on the development of neurological diseases or even cancer, which have been known to be mainly caused by oxidative stress [48,49,50]. Likewise, an increased expression of antioxidant proteins, such as heme oxygenase (HO-1), known to limit inflammatory response, or NQO-1, glutathione reductase, glutathione peroxidase 2, and GST-pi, was also reported [51]. It is noteworthy that BPC 157 has a particular angiogenic effect in wound healing, which may also take place indirectly through the involvement of antioxidant enzymes. Indeed, surely antioxidant enzymes have been known to regulate, for example, processes accompanying muscle regeneration—inducing angiogenesis and reducing fibrosis [51]. In this aspect, BPC’s proangiogenic effect was attributed to the stimulatory effects of vascular endothelial growth factor receptor 2 (VEGFR2) [14].

**Figure 1:**
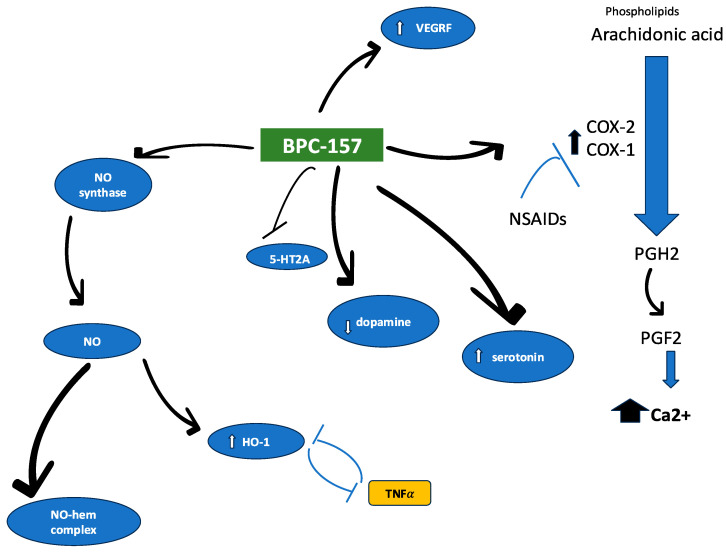
Proposed targets for BPC 157 biological effects. The BPC 157 pentadecapeptide positively interacts with nitric oxide synthase to increase expression of several antioxidants, including heme oxygenase (HO-1). However, on the other hand, NO generated from NOS, apart from its cytotoxic activity, is used in immune responses as well as being crucial in maintaining activity in developing neurons. It can bind directly to the heme iron of NOS to trigger various chemical and biochemical reactions (e.g., NO deoxygenation associated with scavenging of NO and vasoconstriction, as observed for hemolytic diseases [52], or S-nitrosylation of HbA hemoglobin responsible mainly for lung injury associated with an increased formation of red blood cells [53]). It was found to have a modulatory effect on dopamine level, as it antagonizes catalepsy induced by dopamine antagonist haloperidol [54], while mitigating harmful effects caused by amphetamine [55]. Also, the peptide may stimulate VEGF receptors, while VEGF, particularly VEGF-C, has been reported to induce expression of cyclooxygenase COX-2 and vice versa. In turn, PGF2alpha is known for its increasing activity in raising intracellular calcium levels, and therefore it is an important marker of myocardial stress and heart failure. VEGF is also a well-known agent that causes NO release [56]. Abbreviations: COX (1, 2), cyclooxygenase; HbA, hemoglobin; HO-1, heme oxygenase; NO, nitric oxide; NOS, nitric oxide synthase; NSAIDs, non-steroidal anti-inflammatory drugs; PGF, prostaglandin F2alpha; PGH2, prostaglandin H2; TNF-α, tumor necrosis factor; VEGFR, vascular endothelial growth factor receptor; 5HT2A, serotonin receptor type 2A; ↑, increase; ↓, decrease.

Some of the proposed molecular targets of BPC 157, based on the literature data, are presented in Figure 1.

As presented above, the main molecular target for the peptide is NO, particularly eNOS-derived NO, which can act on a number of target enzymes and proteins. Therefore, considering a broad spectrum of effects that may result from the induction of NO synthesis, a deep analysis of its interaction with potent drugs affecting NO pathways should be performed (e.g., aspirin that has been shown to inhibit eNOS [57]). Indeed, increased nitric oxide production is defined with renal vasodilation through cGMP-dependent protein kinase (PKG) activation and natriuresis [58]. Moreover, it is found as a potent inhibitor of platelet aggregation and adhesion to the vascular wall [59]. Hence, its administration should be especially specified in subjects with cardiovascular diseases and/or cardiovascular risk factors. Apart from this, NO signaling is likely to contribute to a variety of neurodegenerative pathologies such as excitotoxicity following stroke, multiple sclerosis, Alzheimer’s, and Parkinson’s diseases [60]. A search of the literature shows numerous other investigations regarding the role of the eNOS enzyme and NO itself, which finally suggest that NO in moderate amounts can be neuroprotective while NO in large concentrations will be neurotoxic or cytotoxic.

The ADME profile (i.e., absorption, distribution, metabolism, excretion) of a substance/drug is one of the crucial elements that enables us to unveil the behavior of a drug under the influence of different processes that take place in our body. It is also an excellent tool to identify and select a proper drug candidate of desired characteristics, e.g., in terms of the degree of binding to plasma proteins, bioavailability, and metabolism. Finally, the ADME optimization of the drug can be useful in some ways to further substantiate its hypothetical efficacy and safety.

Given the widespread popularity of the BPC 157 pentadecapeptide, it becomes important to investigate its pharmacokinetics. In this context, only one study conducted in rats and dogs by He et al. [61] has been presented so far, describing the behavior of the compound during its exposure to the body. The study comprised a single intravenous (i.v.) administration of the peptide (20 μg/kg in Sprague-Dawley rats and 6 μg/kg in beagle dogs), a single intramuscular (i.m.) administration of 20, 100, or 500 μg/kg (rats) and 6, 30, or 150 μg/kg (dogs), and repeated i.m. administrations of 100 μg/kg (rats) and 30 μg/kg in dogs of BPC 157 for seven consecutive days.

According to the results, the mean absolute bioavailability after i.m. injections varied depending on the test animal, reaching levels of approximately 14–19% and 45–51% in rats and beagle dogs, respectively. Both the maximum concentration (Cmax) and the Tmax, at which the highest drug concentration occurred, indicated that BPC 157 reached its maximum as soon as it was eliminated from the plasma in two experimental animal models. Following single i.m. injections of doses of 20, 100, or 500 μg/kg to rats, the peak time (Tmax) of each dose was 3 min, while the Cmax of each dose was equivalent to 12.3, 48.9, and 141 ng/mL, respectively. Similarly, in beagle dogs, BPC 157 reached its maximum after 6.33, 8.67, and 8.17 min with Cmax values of 1.05, 3.30, and 26.1, respectively. In addition, the distribution range for the *i.m.* administered substance (100 µg/300 μCi/kg of radioactive [^3^H]BPC 157) was quite wide in rats, as some of its concentrations were detected in the skin, intestine, lung, myocardium, skeletal muscle, liver, spleen, and body fat, with the highest average concentration found in the kidney. The study showed that the brain also contained the compound, although the concentration was the lowest there. This, in turn, suggests a low ability of BPC 157 to cross the blood–brain barrier (BBB) [61].

**Figure 2:**
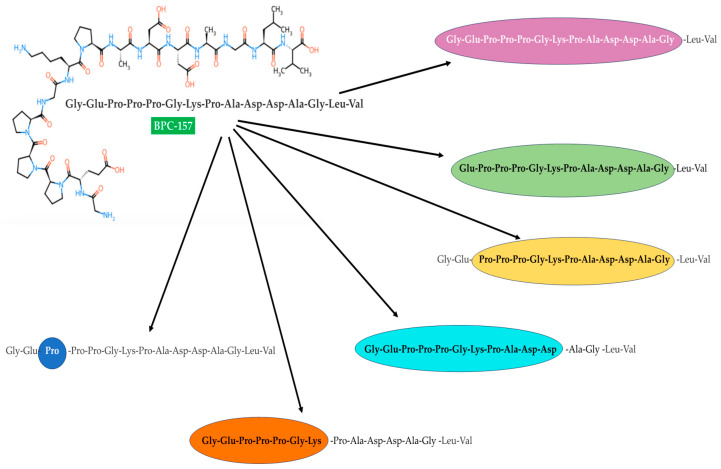
Chemical structures of BPC 157 metabolites as presented by He et al. [61]. Sequences in a colored background represent metabolites.

Importantly, BPC 157 is metabolically degraded into six peptide metabolites, of which proline is the main one and there are five others with the structures shown in Figure 2. It is noteworthy that these six new small-molecule peptidic metabolites were similarly detected in the urine, bile, fecal, and plasma samples.

Finally, BPC 157 was characterized by a rapid degradation; its elimination half-life (t1/2) is below 30 min [61].

The pharmacokinetic profile of BPC 157 was also estimated in humans by Veljaca and colleagues [62] in 2002, but details of these studies are rather scanty.

## 3. Potential Therapeutic Use—Preclinical Studies

### 3.1. Cancer

There are many publications dealing with the peptide as an effective cytoprotective agent. So far, however, there have been none relating directly to cancer, as there is no information on whether BPC 157 can attack cancer cells or not. Of all available scientific publications, only one describes the positive effect of the peptide on general cachexia as a consequence of cancer. In this context, it was shown that BPC 157 at a dose of 10 μg/mL three times a week i.p. significantly improved total body weight in the C26 colon adenocarcinoma-induced cancer cachexia mouse model as compared to that of the control group of animals [15]. In addition, administration of BPC 157 to mice led to attenuation of expression of various pro-inflammatory markers, such as IL-6 and TNF-alpha. Although the tumor volume was lower, there was no statistical significance between the BPC 157-treated and untreated xenograft mice [15].

### 3.2. Pain

Pain is a condition well known to every human being from birth, and it remains an integral part of human existence. To date, it has been demonstrated that opioids, along with NSAIDs, are among the most common and effective analgesic drugs, owing to their rapid and powerful activity. However, long-term activation of an opioid receptor with either chronic use or abuse of opioids leads to highly undesirable and clinically significant side effects. These include nausea and vomiting [63], respiratory depression [64], and subsequently tolerance and dependence [65]. Also, NSAIDs, while lacking addictive potential, can cause a range of complications, such as upper gastrointestinal bleeding [23,66] and renal dysfunction [67,68]. Therefore, there is still the need for the discovery and/or development of a drug candidate characterized by at least a comparable pain-relieving effect to opioids but with a much better safety profile in terms of expected adverse reactions. Intriguingly, BPC 157 could be considered as an alternative to those problems, as it is likely to exhibit such desired characteristics.

BPC 157 has been found to exert antinociceptive activity in a formalin-induced animal inflammatory pain model [69]; however, this is only partial, as it dose-dependently reduced the number of flinches during phase 1 but did not decrease their number during phase 2. Similar results were reported by Jung and co-workers [70] who presented BPC 157 as a compound with moderate efficacy against postoperative incisional pain in rats within a 7-day period. In fact, the peptide was found to attenuate the withdrawal threshold only for a short time post-incision. Unfortunately, this effect vanished after time ranging from 6 h to 4 days.

The expected analgesic effect induced by the peptide has not been studied more extensively, especially in other animal models of pain. However, surprisingly one study indicates a slightly different effect of the compound. Indeed, the pentadecapeptide was found to be ineffective when administered to healthy animals exposed to the hot-plate test maintained at 55 °C. When injected i.p. into mice given morphine, BPC 157 behaved similarly to naloxone, since morphine-induced analgesia was reduced to the levels recorded in the control saline mice [71]. Therefore, definitely, more studies are needed to determine the types of pain for which the compound exerts its analgesia, as well as to identify sites of its activity in the process of pain perception and processing.

### 3.3. Alcohol-Induced Adverse Effects

BPC 157 has also attracted attention in the treatment of various problems associated with alcohol consumption, particularly those related to liver and gastrointestinal tract damage. One of the first reports [72] demonstrating the positive hepatoprotective effect of BPC 157 dates back to 1993, although this was not necessarily related to alcohol exposure. Nevertheless, it was shown that rats administered with the peptide at doses of 1 μg and 10 ng/kg (intraperitoneally i.p. or intragastrically i.g., respectively) prior to carbon tetrachloride (CCl_4_) intoxication showed a significant drop in bilirubin and aspartate aminotransferase as compared to that of the control group. In addition, BPC 157 reduced the severity of lipid accumulation in hepatocytes and the extent of coagulative necrosis after CCl_4_ administration, which were severe in the controls. Of note, pretreatment with somatostatin or bromocriptine used as protective agents against liver damage [72,73,74] had no effect on the symptoms induced by CCl_4_ [72].

In 2001, Prkacin et al. [75] provided new evidence on the activity of BPC 157 as a novel compound capable of preventing, attenuating, and reversing the gastric and liver lesions induced by chronic alcohol drinking. In this context, the animals were divided into three groups that received the peptide (10 mg and 10 ng/kg b.w. i.p. and i.g., respectively) either within 10 days preceding 3 months of alcohol consumption (prophylactic), simultaneously during the entire alcohol drinking period, or throughout the last month of the drinking. In all animal groups, BPC 157 showed efficacy in attenuating gastric lesions and reversing advanced lesions similarly to propranolol and ranitidine used as controls.

Similar findings were reported by Gojkovic et al. [21], who demonstrated that rats injected i.g. with alcohol (96%; 1 mL) and intraperitoneal BPC 157 (delivered 1 min after ethanol administration) showed no changes in liver tissue as compared to animals receiving alcohol as the control. In this case, the control group was characterized by dilated central veins, sinusoids, and blood vessels in the portal tract of the liver, as well as a ballooning of hepatocytes in zone 3 of the liver lobules.

A few years later, in 2004 and 2006, other researchers [9,76] supplemented the previously published study with new results that additionally pointed to the positive effect of the peptide in reducing the physiological and behavioral negative effects triggered by acute or chronic alcohol consumption. For example, preclinical in vivo studies performed by Boban-Blagic et al. [76] showed that the peptide reduced the severity of ethanol withdrawal after chronic treatment (13 days), which manifested itself in the form of tremors and tonic–clonic seizures. The temperature and overall survival rate were also improved.

BPC 157’s protective effects have also been examined in rats that were given ethanol (96%; 1 mL/rat) directly on the tongue and swallowed [77]. Again, in animals treated with the peptide, the pattern of injury that occurred differed significantly from that in controls. Indeed, while in rats exposed to alcohol without BPC 157 administration, more pronounced edema of stroma and striated muscle was seen, in the peptide-treated animals only mild reactive changes on the surface epithelium and scarce, unevenly distributed accumulations of polymorphonuclear inflammatory cells on the muscle were noticed.

### 3.4. Wound Healing and Regeneration

Much work has been expended to demonstrate the potent effect of BPC 157 on the healing process in various experimental models in vivo (i.e., alkali-burn wounds, alloxan-induced gastric lesions) [78,79]. Indeed, this activity of the peptide was the first to be demonstrated, and it remained one of the most adequately described. Therefore, in order not to repeat the information given in several review papers, we refer the reader to some of those papers [80,81]. Nevertheless, a brief summary of healing properties induced by the compound is presented in Table 1.

In this context, BPC 157 has been shown to be effective in curing rat vesicovaginal fistulas, continuous urine leakage through the vagina, and bladder stones [87]. In fact, the peptide either reduced or eliminated spontaneous urine leakage in female animals, accompanied by enhanced epithelialization, collagenization, granulation, and neovascularization, as well as by lowering inflammation, necrosis, and adhesion formation. Finally, toxicity mediated by various types of drugs, such as bupivacaine [88], NSAIDs [89], clopidogrel [90], and even by lead and fluoride [91], as well as by NiCl_2_ and KMnO_4_ [92], was attenuated.

### 3.5. Neuropsychiatric Disorders

Among mental disorders, depression, anxiety, and schizophrenia are the best known and most frequent. All those pathological conditions result from disturbances in the levels of various brain neurotransmitters such as serotonin (5-hydroxytryptamine (5-HT)) and dopamine. In major depressive disorder (MDD) and other depression-like conditions, it is still hypothesized that serotonin concentration, which is abnormally reduced, is the main factor [93]. However, as Moncrieff and colleagues point out in an attractive systemic review, there is no convincing evidence that depression could be associated with or caused by lower serotonin concentrations or activity [94]. With anxiety or schizophrenia, dopamine levels are elevated [95,96]. However, other neurotransmitters and receptor systems are also involved (e.g., γ-aminobutyric acid (GABA) and serotonin) [97,98]. Nevertheless, most first-line drugs for the treatment of those disorders target the serotonin and/or dopamine systems either directly (e.g., in depression) or indirectly via modulation of GABA-A receptors.

In 2004, it was reported that subcutaneous (s.c.) administration of the BPC 157 peptide to rats (10 μg/kg/day for 7 days) resulted in brain region-specific increases in serotonin synthesis [99], as determined by the precise alpha-[^14^C]methyl-L-tryptophan (alpha-MTrp) autoradiographic method. At the same time, the study was preceded by another one conducted by Sikiric et al. [100], who in 2000 showed that BPC 157 can serve as a potent antidepressant as measured by the Porsolt test (where peptide counteracts freezing). In addition, it was found that BPC 157 effectively reduced some of the symptoms occurring in the serotonin syndrome [26], thus exhibiting a rather specific counteraction of an excessive stimulation of 5-HT receptor subtypes (5-HT2A rather than 5-HT1A). This was demonstrated by the fact that administration of the peptide counteracted 5-HT receptor-dependent symptoms induced by pargyline + L-tryptophan, i.e., hyperthermia and wet dog shake related to 5-HT2A agonism, but not 5-HT1A-related forepaw treading, hind limb abduction, or hypothermia.

Other studies also clearly confirm its interactions with the dopaminergic system. In this context, BPC 157 was found to antagonize the anxiety behavior triggered by amphetamine, whose activity and effects were due to elevated extracellular dopamine levels [55,101]. Similarly, it alleviates withdrawal symptoms in animals chronically fed with diazepam [102], as upon withdrawal, the inhibitory influence of neurons was thought to be suppressed. This, in an effort to “compensate” for previous suppression of the release of DA, taking place during benzodiazepine administration, resulted in a sudden increase in DA concentrations [103].

## 4. BPC 157 in Humans—Clinical Trials and Current Use in Clinical Practice

To date, BPC 157 pentadecapeptide has not been prescribed as a drug, though it is widely accessible on the black market. This is true despite the fact that most of the studies presenting its consistently positive effects were performed on animal models, particularly rodents, while human studies are scarce.

In fact, few clinical studies were conducted to estimate the therapeutic effects of BPC 157. One of the examples is a retrospective study on 12 patients with knee pain who had an intra-articular injection of the peptide into their knees [104]. As a result, it was indicated that in 11 of the 12 subjects, a significant relief in the knee pain was noticed. However, results of the studies are not overly informative and reliable, as there was no survey tool by which the level of improvements could have been defined.

In 2015, a Phase I clinical trial conducted on 42 healthy volunteers (both sexes, aged 18–35 yrs.) was started. This study aimed to determine the safety and pharmacokinetic profile of the BPC 157 peptide (NCT02637284) [105]. Unfortunately, in 2016, the researchers cancelled submission of the results.

Considering the scarcity of human studies on the peptide and the fact that not all studies conducted on animals can always be freely transferred to humans, more extensive human-oriented studies indicating therapeutic and toxic profiles of BPC 157 are required at once. This also includes the need to compare different methods of drug administration in one model, such as oral vs. intraperitoneal and others, in order to establish the effects and make the results more reliable.

## 5. BPC 157 and Probable Toxicity

With the increasing interest in the BPC 157 peptide and its application potential, the need to define the risk of probable side effects is also escalating. As already mentioned, the peptide exerts pleiotropic effects via different signaling pathways. Intriguingly, there is only insufficient information about its potential side effects, thus making the compound’s effects still unknown. However, as the mechanism associated with its biological activity appears to be regarded as being complex and diverse, this could pose a serious risk for BPC 157 unverified/untested but possible adverse reactions. Hence, we attempted to predict some of the expected adverse consequences of the peptide’s multifunctionality; some examples are presented below.

### 5.1. Angiogenesis Consequences

As BPC 157 has been found to stimulate angiogenesis [14,85], this is likely to have some undesirable consequences.

Angiogenesis is a well-known four-stage process of new blood vessel formation that occurs in healthy individuals (e.g., during the menstrual cycle or muscle growth) but also in immune diseases [106]. Although the proper development of the vascular network enables gas exchange and transport of metabolic products in the organs, angiogenesis also occurs in many pathological conditions [107]. This also applies to cancer, as the formation of blood vessels during carcinogenesis enables oxygen supply to the tumor and enhances its proliferation, diffusion, and metastasis [108,109,110]. Importantly, it has been reported that the pentadecapeptide affects signaling of one of the crucial angiogenic factors, the vascular endothelial growth factor and its receptors (VEGFR). Indeed, BPC 157 increased expression of the VEGFR-2 receptor in rats with hind limb ischemia and in endothelial cell cultures [14,111]. However, the VEGF family and its receptors, including VEGFR, have been found to be expressed in about half of the human cancers studied, such as ovarian cancer, melanoma, thyroid cancer, and more [111,112,113,114].

Similarly, NO that has been reported to be stimulated by the peptide [115] through the impact on endothelial NO synthase (eNOS) has also been known for its stimulatory role in angiogenesis and mediating activity of various angiogenic molecules [116,117,118]. In addition, the expression and activity of NOS correlate with the growth and aggressiveness of human tumors [116,119,120].

Angiogenesis can also be mediated by the promoter of early growth response-1 EGR-1 [121,122]. As BPC 157 revealed its beneficial effects when used to treat full-thickness excisional wounds in genetically diabetic mice, it has been speculated that this activity results from the impact of the peptide on the expression of the immediate response gene (*egr-1*). Also, stimulation of its corepressor nerve growth factor 1-A binding protein-2 (nab2) in Caco-2 cells was noticed [83]. Of note, high expression of EGR-1 is associated with either cardiovascular pathological processes or hepatic injury. Indeed, EGR-1 was found to contribute to the pathogenesis of atherosclerosis [123], stenosed calcific valvular disease [124], and cardiac hypertrophy [125,126]. Furthermore, some reports demonstrate enhanced EGR-1 mRNA expression in the brain after global cerebral ischemia [127]. Additionally, elevated EGR-1 levels can also affect tumor progression, its size, prognosis, and malignancy [122,127,128]. For example, with prostate or gastric cancer, expression of the above-mentioned is higher than that in surrounding tissues [121,122].

This stimulation of angiogenesis by the peptide, and thus the absence of a beneficial effect on tumor growth, was to some extent confirmed by the study of Kang et al. [15], which indicated that administration of BPC 157 to mice with implanted cancer cells did not result in a spectacular reduction in tumor size.

Hence, the use of BPC 157 may not be the right choice, especially in situations where we are not aware of the presence of cancer cells in our body. Since vascular growth is not only up-regulated but also accompanied by significant changes in their architecture (i.e., abnormal vasculature, disorganization, etc.) and function [129,130], a tumor burdened with those vessels may eventually be resistant to various therapeutic methods, including systemic treatment in the form of chemotherapy or radiotherapy [131].

### 5.2. The Role of BPC 157 Metabolites

As just mentioned (please see Section 3), one of the presumable metabolites of BPC 157 is proline (Figure 2) [61]. Free endogenous proline was found to play an important role in oxidative stress. A proper example is a study of Krishnan and colleagues [132], who reported a dose-dependent proline-induced protection of intracellular glutathione levels in HEK 293 cells exposed to H_2_O_2_. However, in humans, proline has been known to be catalyzed by proline oxidase (POX), being regulated by age or by the peroxisome proliferator-activated receptor gamma (PPARγ) regulator [132,133,134,135]. While its overexpression is associated with the accumulation of reactive oxygen species resulting in cell death [136,137,138,139]. Moreover, POX activity can result in immediate oxidative changes in the cellular environment by simultaneously depleting intracellular proline and producing superoxide (O_2_^•−^) [137]. This just-formed superoxide, together with its reduced metabolites, can interact with a great number of different naturally occurring body biomolecules, at least partially leading to the development of many diseases such as cardiovascular disease, cancer, chronic inflammation, dementia, and amyotrophic lateral sclerosis, among others [140,141]. Superoxides have also been demonstrated to be an underlying cause of cytotoxicity mediated by their interactions with NO to form peroxynitrite (ONOO^−^/ONOOH) [142,143]. On the other hand, superoxide formation contributes to reduced NO bioactivity [144], which can also have undesirable pathological effects such as high blood levels, type 2 diabetes, etc. [145,146].

Therefore, again, there is no convincing certainty that BPC 157 is harmless because, as just mentioned, some of the probable metabolites can be responsible for various adverse effects or trigger a cascade of other events leading directly or indirectly to possible side effects.

### 5.3. BPC 157 Stimulatory Effect on the NO System

Based on several studies, it was demonstrated that BPC 157 stimulates the NO system. However, since NO mediates various widespread processes in the cell, an increase in NO beyond the optimum level can lead to some undesirable processes. A good example is its impact on heme metabolism. In fact, NO at high levels inhibits heme insertion in hemoglobin, NO synthases, and heme thiolate enzymes, such as cytochrome P450’s (CYP) in different manners (i.e., through the direct binding to the heme iron or to covalent modifications in heme transfer proteins) [147,148,149]. However, the so-called “heme-anemic” proteins with an incomplete heme saturation naturally occur outside the circulatory system [150]. Nevertheless, modification of heme-containing molecules can affect our health. Iron deficiency, followed by the absence of heme in hemoglobin, induces anemia. Similarly, diminished CYP heme results in its low activity to alter, among other things, drug and xenobiotic metabolism. In addition, while BPC 157 increases NO concentration under inflammation, this can initiate neurodegenerative states such as Alzheimer’s or Parkinson’s diseases, in particular via iron accumulation in the brain [151,152,153]. Other reports indicate that excessive levels of NO, as a result, for instance, from BPC 157 activity, can be responsible for damage of mitochondrial iron–sulfur enzymes and hence for inhibition of mitochondrial respiration through inhibition of cytochrome oxidase [154,155]; this is also true for mitochondrial complex I (or NADH-ubiquinone oxidoreductase) and III (or ubiquinol–cytochrome *c* oxidoreductase) [156,157]. The inhibition of the mitochondrial electron transfer chain by NO leads to the formation of high levels of a strong oxidant peroxynitrite [158]. This, in turn, especially at extremely high concentrations, has been noticed to induce modifications of different molecules, including DNA [159] and proteins [160], which ultimately can be one of the major contributors in the pathogenesis of various neurodegenerative diseases [161].

## 6. The Future of BPC 157

The broad spectrum of beneficial effects exerted by BPC 157 has given rise to various ideas on how to improve the peptide in terms of its administration depending on the circumstances or to improve the pharmacotherapy of diseases through the simultaneous use of the molecule with other clinically available drugs. This trend has clearly been evident in the patent literature (Table 2).

An interesting example of the probable use of the BPC 157 pentadecapeptide was presented in the US patent by Bentz et al. [162]. This invention aims to demonstrate the efficacy of a combination of an antidiabetic incretin drug, semaglutide, known for its weight-reducing effect, and a gastric peptide BPC 157. It was hypothesized that this mixture produces a stronger physiological response in the body, probably owing to synergistic interactions between the two components, or else that it reduces any side effects of semaglutide (i.e., nausea, unusual fatigue and weakness, indigestion, etc.).

Other properties of BPC 157, in particular the anti-inflammatory activities, were utilized and presented in 2021 and 2023 [163,164] and earlier in 1998 [19]. In the first two cases, it was suggested that the peptide could serve as an effective adjuvant for a clinical drug to either prevent or treat acute respiratory distress syndrome (ARDS) that was frequent during the COVID-19 pandemic. However, other circumstances can also be responsible, such as viral pneumonia, sepsis, chest injuries, burns, blood transfusions, aspiration of gastric contents, pancreatitis, intravenous drug use, abdominal trauma, and acute radiation syndrome [165,166,167].

In contrast, another invention presents the use of BPC 157 in the treatment of multiple sclerosis (MS) in combination with corticosteroids [168]. This proposal has a strong argumentation because in addition to the anti-inflammatory effect of BPC 157, it was also thought to have some benefit in the treatment of depression, muscle weakness, etc., or the common symptoms of MS [169,170]. However, the inventors also suggested that the peptide could be used not only to treat MS itself, either alone or with concomitant corticosteroid therapy, but also to prevent relapse or to treat MS in remission.

Interestingly, the healing capabilities of BPC 157 have also been utilized in still another US patent [171]. This discovery relates to the simultaneous use of the peptide with at least one of the umbilical cord/placental blood, plasma, mesenchymal stem cells (MSCs), and/or mono-nucleated cells (MNCs) in the treatment of ocular diseases, including dry eye, inflammation, oxidative stress, and eye injury). The combination can also consist of hyaluronic acid or any therapeutic agent used.

The properties offered by the compound have also been exploited by a Chinese group, who presented BPC 157 as an interesting component of medical dressings for scar repair, which is effective in shortening the time for wound healing [172]. Based on the experiments performed, the authors indicated that changing the amount of BPC 157 used in the formulation affects the bacteriostatic properties of the medical dressing. Furthermore, as BPC 157 was mixed with various compounds such as allopurinol, a cooperation between both chemicals was observed. This resulted in the effective formation of a protective layer for the wound surface, the reduction in the evaporation of water on the surface of the scar, and the increase in the water content of the skin cuticle, so that the scar after the wound surface is healed was found to be repaired well.

Another patent describes BPC 157 as a potent therapeutic component of topical preparations for pain management and injury rehabilitation [173], while others show efforts to produce a single oral dosage form as a capsule-in-capsule system with the use of BPC 157 [174]; this aims at protecting the peptide active agents from proteolytic degradation and the action of GI acid.

**Table 2:** Sample of patents with BPC 157 utilization.

No.	Patent Title	Inventors	Patent Number	Ref.
1	Fibroblast mediated expansion and augmentation of immune regulatory cells for treatment of acute respiratory distress syndrome (ards)	Ichim, T.; O’Heerin, P.	US 2023/0141224	[163]
2	Peptides and adjuvants for augmentation of fibroblast therapy for coronavirus	O’Heeron, P.; Ichim, T.	WO 2021/202031	[164]
3	Medical dressing for repairing scars and preparation method thereof	Zhejiang Top Medical Medical Dressing Co., Ltd.	CN 2024/118615479	[172]
4	New BPC peptide salts with organo-protective activity, the process for their preparation and their use in therapy	Sikiric, P.; et al.	WO 1998/052973	[19]
5	Sublingual semaglutide-BPC 157 combination for weight loss	Bentz, S.; Lucht, A.; Kocher, S.	US 2023/11833189	[162]
6	Usefulness of pentadecapeptide for the treatment of multiple sclerosis	Bota, B.	Croatia Patent 2013/1075	[168]
7	Formulation and treatment for ophthalmic disorders	Vitti, P.R.	US Patent 2022/0249575	[171]
8	Systems and methods for treating persistent pain of neurogenic origin and complex injury	George, D.	WO 2021/252292	[173]
9	Pharmaceutical single dosage form for oral delivery of peptides.	Majewski, F	EP 2022/4226918	[174]
10	Compositions for improving health	Crisler, M	WO 2024/073762	[175]

Although the number of patent applications relating to the therapeutic use of the BPC 157 peptide is modest to date, it can be expected that this interest will increase in the future. An obstacle here, however, is the still-unknown safety profile of the molecule. Hence, further preclinical studies to systematize knowledge of its expected side effects or interactions can be a key element in opening the door to new options.

## 7. Conclusions

The majority of papers on BPC 157 pentadecapeptide affirm the enormous interest in this substance; it can undoubtedly be said that it is one of the most comprehensively investigated compounds today. Importantly, almost every original publication points to its immense potential. BPC 157 is therefore worth investigating for future clinical applications. However, given the proposed molecular pathways and targets, it is of importance to focus on probable adverse effects resulting from its single or chronic administration before falling into complete admiration and considering the compound as a panacea effective for all conditions with a highly postulated safety profile. In this context, for instance, the pharmacokinetic profile needs to be defined in detail, including the type of metabolic enzymes involved in its degradation, interactions resulting from the binding of the drug to plasma proteins, etc. This should also concern an in-depth analysis of the mechanism of action, including NO as the target most affected by the peptide, as well as the off-targets and their possible effects. Another important issue should also be addressed. Although the peptide is derived from human gastric juice, there are no completed clinical studies describing its efficacy in humans. It should be borne in mind that there are obvious differences between the physiologies of rodents and humans. Therefore, a still completely unknown mechanism of action, efficacy, and safety profile cannot be ignored. As can be seen in the literature on the peptide BPC 157, most, if not all, studies are limited to small animal models (i.e., rats and mice), and the routes of administration are also limited to a small number, making it impossible to determine the overall efficacy of the drug. Finally, studies should be carried out on the potential toxicity of the drug, and more attention must be paid to possible side effects (sometimes the simplest ones, such as weight loss, increase/decrease in body temperature, etc.) that may occur when the substance is used in a given disease model.

## Figures and Tables

**Table 1:** Examples of BPC 157-exerted healing effects in different preclinical experimental models.

Preclinical Model	Dose Regiment	Species	Outcomes Efficacy	Ref.
Aloxan-induced diabetic wound	10, 100, 500, and 1000 g/0.05 g of carbopol gel daily for 5 days (s.c.)	Male Wistar Han rats	Promotion of mature collagen content in granulation tissue	[82]
Excisional, non-occulated full-thickness wound	100 μg/wound once daily for 18 days	Genetically diabetic female C57BL/KsJ db^+^/db^+^ mice	Immediate closure of wounds; impact on the organization of collagen; stimulation of granulation tissue formation	[83]
Thermal (flame) burn-induced wound	Topically 1 μg/1 g of vehicle (commercial neutral cream)	NMRI-Hannover male mice	Improvement in all parameters of burn healing (i.e., less edema, reduction in the number of inflammatory cells, advanced formation of dermal reticulin and collagen fibers)	[79]
Skin alkali burn	200–800 ng/mL of the drug applied topically twice every day for 18 days	Male Sprague-Dawley rats	Fast wound closure (i.e., impact on the organization of granulation tissue formation, re-epithelialization, and dermal remodeling	[84]
Muscle crush injury model	10 μg/kg i.p. once a day for 13 days	Male Wistar Albino rats	Increased blood vessel formation	[85]
Animal’s tail amputation	10 μg/kg or 10 ng/kg i.p. before amputation	Male Wistar Albino rats	Reduction in bleeding time and blood loss vs. saline-treated animals; attenuation of acute thrombocytopenia	[86]
